# Anti-Aging and Anti-Inflammatory Effects of Compounds from Fresh *Panax ginseng* Roots: A Study on TNF-α/IFN-γ-Induced Skin Cell Damage

**DOI:** 10.3390/molecules29225479

**Published:** 2024-11-20

**Authors:** Minseo Kang, Somin Park, So-Ri Son, Yedam Noh, Dae Sik Jang, Sullim Lee

**Affiliations:** 1 Department of Life Science, College of Bio-Nano Technology, Gachon University, Seongnam 13120, Republic of Korea; bana825@gachon.ac.kr; 2 Department of Biomedical and Pharmaceutical Sciences, Graduate School, Kyung Hee University, Seoul 02447, Republic of Korea; somin0915@khu.ac.kr (S.P.); allosori@khu.ac.kr (S.-R.S.); dam6121@khu.ac.kr (Y.N.)

**Keywords:** ginsenoside Rf, *P. ginseng*, anti-aging, anti-inflammatory, ROS, MMP-1, skin barrier, TNF-α/IFN-γ

## Abstract

*Panax ginseng* (Korean ginseng) is renowned for its health-promoting properties, attributed to its bioactive compounds, including saponins, polyphenols, and polysaccharides, which possess both antioxidant and anti-aging activities. This study investigated the anti-aging and anti-inflammatory effects of compounds isolated from the hot water extract of fresh *P. ginseng* roots, evaluating their resistance to TNF-α/IFN-γ-induced skin cell damage. Among 14 compounds, ginsenoside Rf (compound **2**) showed significant multi-target effects. In NHDFs, ginsenoside Rf and others effectively reduced intracellular ROS, demonstrating strong antioxidant properties. Additionally, they inhibited MMP-1 expression, a key enzyme in collagen degradation, and promoted pro-collagen Type I synthesis, countering the negative effects of TNF-α and supporting skin health. Further analysis showed that ginsenoside Rf reduced the secretion of inflammatory cytokines like IL-1β and IL-6, exhibiting anti-inflammatory effects. It also promoted the expression of crucial skin barrier proteins, including LOR, AQP3, FLG, and KRT1 in TNF-α/IFN-γ-stimulated NHEKs, enhancing skin hydration and structural integrity. These results suggest that compounds from *P. ginseng* roots, especially ginsenoside Rf, hold promise as skincare agents targeting skin aging and inflammation. Future research should further explore their mechanisms and optimize their applications in dermatological treatments.

## 1. Introduction

Human skin is composed of multiple layers, including the outermost epidermis and the underlying dermis. The epidermis, being a stratified layer, undergoes a maturation process that enables the skin to serve as a robust physical barrier, safeguarding internal tissues from external threats. Beneath the epidermis, the dermis comprises an intricate extracellular matrix (ECM), predominantly made up of collagen and elastin fibers [1]. These essential proteins, synthesized and secreted by skin fibroblasts, play a crucial role in maintaining skin elasticity and structural integrity. However, during the aging process, the components of collagen and elastin are progressively degraded, contributing to visible signs of skin aging.

Skin aging can be categorized into two distinct types: endogenous and exogenous aging. Endogenous aging is an intrinsic process driven by factors such as cellular senescence and a diminished capacity for DNA repair within cells [2]. As cells age, their ability to divide and repair damage decreases, leading to gradual tissue deterioration. Conversely, exogenous aging is predominantly influenced by environmental factors, with photoaging, resulting from exposure to ultraviolet (UV) radiation, considered the primary cause. UV light exposure triggers the release of cytokines, including tumor necrosis factor-alpha (TNF-α), which in turn elevates the production of reactive oxygen species (ROS). The increase in intracellular and extracellular ROS levels induces oxidative stress, which accelerates skin aging, marked by wrinkles, sagging, and other visible signs [3,4].

The process of skin aging, particularly through UV radiation (UVR), is largely mediated by the generation of photochemically reactive oxygen species. These include hydrogen peroxide (H_2_O_2_), superoxide ions (O_2_^−^), hydroxyl radicals (OH), and singlet oxygen (H∙) [5]. Excessive ROS levels from UV exposure lead to the upregulation of matrix metalloproteinase-1 (MMP-1), an enzyme that degrades collagen, as well as a reduction in collagen synthesis. Elevated MMP-1 activity results in the breakdown of the structural proteins that form the skin’s extracellular matrix. As the integrity of this matrix diminishes, accumulated skin damage becomes more pronounced, leading to the development of wrinkles and other characteristics of photoaged skin [6,7]. Thus, the disruption of collagen integrity by UV-induced oxidative stress plays a pivotal role in the progression of skin aging.

The emphasis on anti-aging has become a fundamental aspect of maintaining skin health, driving the development of strategies aimed at preserving youthful and resilient skin. In line with this, there is a growing trend toward the use of natural products, especially those derived from plants, which are increasingly favored for their environmentally friendly and sustainable properties. In this study, we conducted a thorough evaluation of various compounds obtained from the fresh roots of *Panax ginseng* (Korean ginseng) to assess their potential anti-aging properties. The focus was on investigating how these natural compounds could mitigate the signs of skin aging, contributing to healthier, more youthful skin.

*Panax ginseng* C.A. Meyer (Araliaceae) is a well-known medicinal plant that is distributed across 64 countries, with its primary cultivation and usage concentrated in South Korea and China [8]. The roots of *P. ginseng* have been extensively utilized as core ingredients in various health supplements due to their broad spectrum of biological activities, which include notable antioxidant, anti-inflammatory, and homeostatic effects [9]. Numerous pharmacological studies have confirmed that *P. ginseng* exerts protective effects against skin aging by mechanisms such as reducing inflammation and stimulating collagen synthesis, as demonstrated through both in vitro and in vivo experimental models [10,11,12,13,14].

Among its diverse bioactive components, ginsenosides are recognized as the key agents responsible for the majority of *P. ginseng*’s beneficial biological properties, including its anti-aging effects on the skin [15]. Specific ginsenosides, such as ginsenoside Rg2 and compound K, have shown significant anti-aging activities in cell models like Hs68 fibroblasts and UV-irradiated NIH3T3 cells, respectively. These effects were mainly correlated with the inhibition of matrix metalloproteinase-1 (MMP-1) expression, which helps to prevent collagen degradation and preserve skin structure [16,17]. While ginsenosides have been the primary focus of research, it is important to note that *P. ginseng* also contains various phenolic compounds and lignans. However, their effects on skin aging have not been explored as thoroughly, with only limited studies available on their potential roles [18,19]. Given these gaps, the present study focused on isolating and identifying bioactive compounds in ginseng that have not yet been extensively reported, to evaluate their efficacy and further assess their potential to mitigate skin aging and inflammation.

## 2. Results

### 2.1. Isolation and Structure Identification of Compounds ***1**–**14*** from the Hot Water Extract of P. ginseng Roots

**Figure 1:**
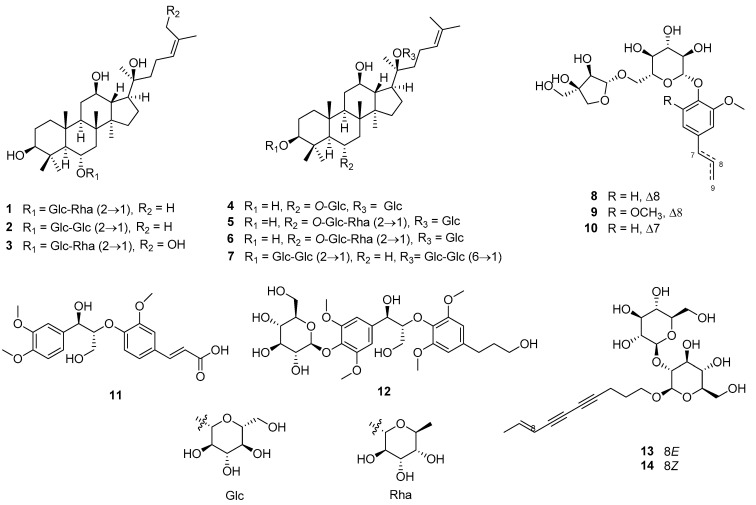
Isolated compounds **1**–**14** from the hot water extract of fresh *P*. *ginseng* roots.

The structures of known compounds were identified as ginsenoside Rg2 (**1**) [20], ginsenoside Rf (**2**) [21], panajaponol A (**3**) [22], ginsenoside Rg1 (**4**) [23], ginsenoside Re (**5**) [21], 20-glucoginsenoside Rf (**6**) [24], ginsenoside Rb1 (**7**) [21], eugenyl *O*-β-apiofuranosyl-(1″→6′)-*O*-β-glucopyranoside (**8**) [25], 2,6-dimethoxy-4-(2-propen-1-yl)phenyl 6-*O*-d-apio-β-d-furanosyl-β-d-glucopyranoside (**9**) [25], 2-methoxy-4-(1*E*)-1-propen-1-ylphenyl 6-*O*-d-apio-β-d-furanosyl-β-d-glucopyranoside (**10**) [20], *threo*-guaiacylglycerol-β-ferulic acid ether (**11**) [26], symplocosneolignan A (**12**) [27], 8*E*-decaene-4,6-diyn-1-*O*-β-d-glucopyranosyl-(1″→2′)-β-d-glucopyranoside (**13**) [28], and panaxjapyne D (**14**) [29] by comparing their NMR spectroscopic data with those from published data (Figure 1). In addition, compounds **11**–**14** have not been isolated from *P. ginseng* in previous research.

### 2.2. Effects of the Hot Water Extract and Compounds ***1**–**14*** on Intracellular Reactive Oxygen Species (ROS) Secretion in NHDFs

**Figure 2:**
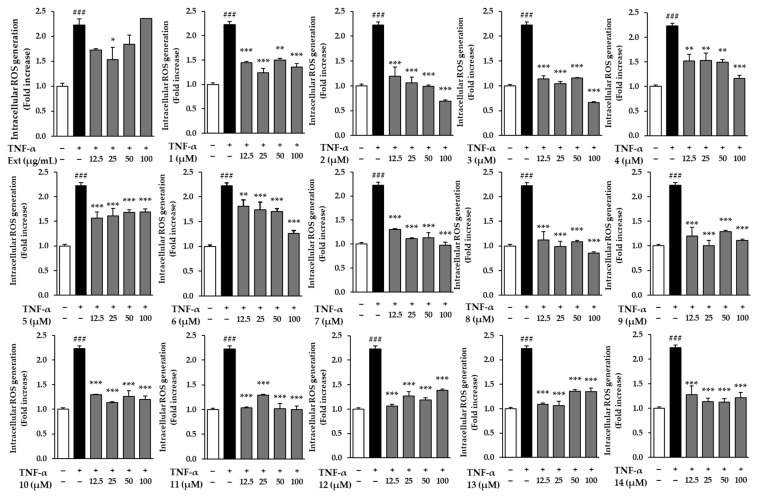
Effects of the hot water extract and compounds **1**–**14** on intracellular ROS accumulation. Intracellular ROS levels are expressed as a percentage relative to vehicle control. Data represent the mean ± standard error of the mean (SEM) from three independent experiments. Statistical significance is indicated as ^###^ *p* < 0.001 vs. vehicle control, and * *p* < 0.05, ** *p* < 0.01, and *** *p* < 0.001 vs. TNF-α-stimulated control.

We evaluated the effects of compounds **1**–**14** on TNF-α-induced damage in NHDFs, first testing cell viability to ensure safety. None of the compounds showed cytotoxicity at the tested concentrations (Figure S1). Based on these results, we selected 100 μM for further experiments to assess their efficacy, ensuring no influence from cytotoxicity. As shown in Figure 2, all compounds exhibited significant effects in reducing intracellular ROS levels in NHDFs treated with TNF-α (*p* < 0.05). TNF-α stimulation led to a marked increase in ROS accumulation, confirming the induction of oxidative stress. However, while the extract did not exhibit a notable effect in reducing ROS levels, all the tested compounds significantly decreased ROS accumulation compared to the TNF-α-stimulated control.

Notably, compound **2** (ginsenoside Rf) was particularly effective, reducing ROS levels to near those of the untreated control group. This suggests that these compounds were able to almost completely counteract the oxidative stress induced by TNF-α. Compound **2** showed strong antioxidant effects, suggesting it could be effective in reducing oxidative damage. Overall, all tested compounds effectively inhibited ROS accumulation, indicating their potential as treatments for managing oxidative stress-related conditions.

### 2.3. Effects of the Hot Water Extract and Compounds ***1**–**14*** on MMP-1 and Pro-Collagen Type Ι α1 Protein Secretion in TNF-α-Treated NHDFs

**Figure 3:**
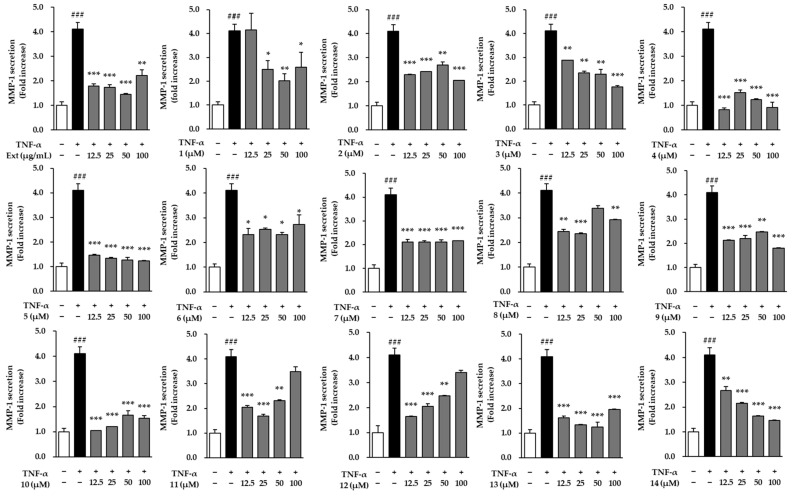
Effects of the hot water extract and compounds **1**–**14** on MMP-1 secretion. MMP-1 secretion in the cell supernatants was measured using an ELISA kit and is presented as fold increases relative to the control. Data were obtained from two independent experiments and are expressed as the mean ± SEM. Statistical significance is indicated as ^###^ *p* < 0.001 vs. vehicle control, and * *p* < 0.05, ** *p* < 0.01, and *** *p* < 0.001 vs. TNF-α-stimulated control.

As shown in Figure 3, the extract and compounds **1**–**14** were evaluated for their ability to modulate matrix metalloproteinase-1 (MMP-1) levels in NHDFs treated with TNF-α. TNF-α stimulation significantly increased MMP-1 expression to 4.10 ± 0.27-fold (*p* < 0.001), indicating enhanced matrix degradation, a typical marker of tissue breakdown. However, both the extract and 14 compounds demonstrated efficacy in reducing MMP-1 levels compared to the TNF-α-stimulated control. The results indicate their potential anti-inflammatory and anti-aging effects.

Notably, extract treatment significantly reduced MMP-1 levels at varying concentrations. Specifically, reductions were observed by 1.79 ± 0.00, 1.73 ± 0.10, 1.45 ± 0.04, and 2.22 ± 0.20-fold at concentrations of 12.5, 25, 50, and 100 μg/mL (*p* < 0.001, *p* < 0.01), respectively, bringing MMP-1 levels closer to those seen in the untreated control group. This shows that the extract effectively inhibits the increase in MMP-1 expression caused by TNF-α.

Among the individual compounds, compounds **2**, **4**, **5**, **7**, **10**, and **13** exhibited the most significant inhibitory effects on MMP-1. Compound **2** (ginsenoside Rf) notably decreased MMP-1 expression by 2.29 ± 0.03, 2.42 ± 0.00, 2.69 ± 0.13, and 2.05 ± 0.01-fold at 12.5, 25, 50, and 100 μM, respectively (*p* < 0.001). In particular, compound **5** (ginsenoside Re) reduced MMP-1 expression by 1.46 ± 0.03, 1.33 ± 0.04, 1.26 ± 0.10, and 1.22 ± 0.03-fold at concentrations of 12.5, 25, 50, and 100 μM (*p* < 0.001). Similarly, compound **10** (2-methoxy-4-(1*E*)-1-propen-1-ylphenyl 6-*O*-d-apio-β-d-furanosyl-β-d-glucopyranoside) also displayed substantial reductions, with MMP-1 levels decreasing by 1.05 ± 0.00, 1.20 ± 0.00, 1.66 ± 0.17, and 1.53 ± 0.12-fold at the same concentrations (*p* < 0.001).

Overall, the findings indicate that while TNF-α stimulation induces a marked increase in MMP-1, both the extract and several individual compounds were effective in inhibiting this upregulation. The notable reduction observed with the extract, as well as compounds **4**, **5**, **10**, and **13**, underscores their potential therapeutic benefits in mitigating collagen degradation and reducing tissue damage associated with inflammatory responses.

**Figure 4:**
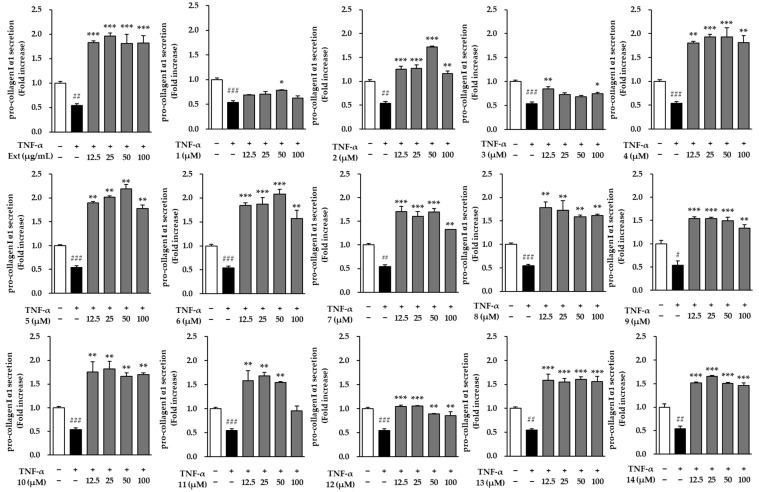
Effects of the hot water extract and compounds **1**–**14** on pro-collagen Type I α1 secretion. Pro-collagen Type I α1 secretion in the cell supernatants was measured using an ELISA kit and is presented as fold increases relative to the control. Data were obtained from two independent experiments and are expressed as the mean ± SEM. Statistical significance is indicated as ^#^ *p* < 0.05, ^##^ *p* < 0.01, and ^###^ *p* < 0.001 vs. vehicle control, and * *p* < 0.05, ** *p* < 0.01, and *** *p* < 0.001 vs. TNF-α-stimulated control.

Figure 4 illustrates the effects of the extract and compounds **1**–**14** on pro-collagen Type I α1 secretion in normal human dermal fibroblasts (NHDFs) treated with TNF-α. TNF-α stimulation significantly reduced pro-collagen secretion (0.60 ± 0.01-fold, *p* < 0.01), highlighting its inhibitory effect on collagen synthesis. However, treatment with 12 compounds, particularly compounds **2**, **3**, **7**, **10**, and **12**, restored pro-collagen levels, suggesting the possibility that they could offset the effects of TNF-α and promote collagen production. In contrast, the extract did not show a significant effect on enhancing pro-collagen secretion at any of the tested concentrations.

Specifically, compound **2** (ginsenoside Rf) demonstrated a significant increase in pro-collagen Type I α1 levels at all tested concentrations. Specifically, it elevated pro-collagen secretion to 0.74 ± 0.04, 0.80 ± 0.01, 0.85 ± 0.03, and 0.80 ± 0.04-fold at concentrations of 12.5, 25, 50, and 100 μM, respectively (*p* < 0.05, *p* < 0.01). These findings indicate that compound **2** effectively mitigates the inhibitory effects of TNF-α on collagen synthesis, thereby supporting pro-collagen production in NHDFs. Similarly, compound **12** (symplocosneolignan A) showed a strong effect at multiple concentrations (*** *p* < 0.001 at 12.5, 25, 50, and 100 μM), demonstrating consistent and potent enhancement of pro-collagen secretion across the tested doses.

Overall, the results indicate that while TNF-α markedly reduces pro-collagen synthesis, several individual compounds were effective in counteracting this inhibition, but the extract was not. Compounds such as **2**, **3**, **7**, **10**, and **12** highlight their potential therapeutic benefits in promoting collagen production, making them promising candidates for addressing conditions where collagen synthesis is impaired.

### 2.4. Spider Chart for Evaluating the Efficacy of the Hot Water Extract and Compounds ***1**–**14*** in TNF-α-Treated NHDFs

**Figure 5:**
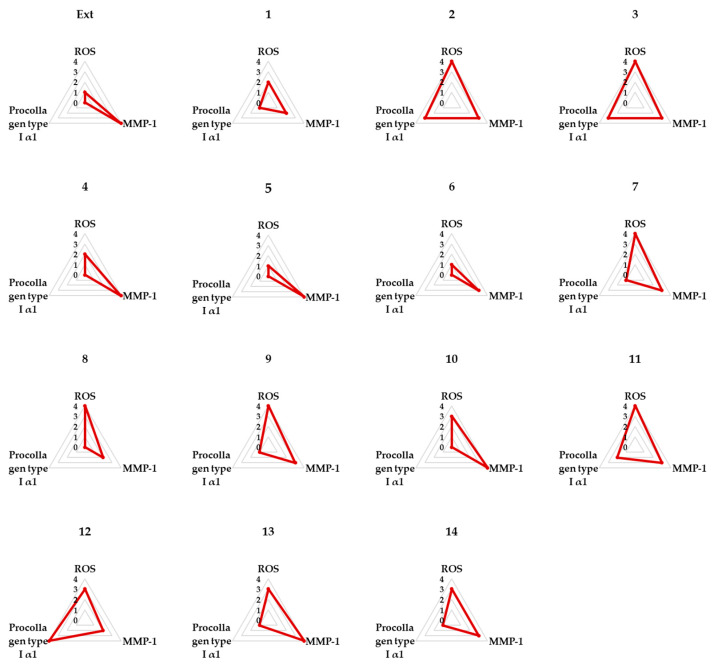
Spider chart comparing the anti-aging efficacy of the hot water extract and compounds **1**–**14**.

The Spider charts in Figure 5 provide a comparative overview of the effects of the extract and compounds **1**–**14** on three critical biological parameters: ROS, pro-collagen Type I α1, and MMP-1 levels in TNF-α-stimulated NHDFs.

The data show that the extract had limited effects. In contrast, compound **2** (ginsenoside Rf) demonstrated strong multi-targeted activity. They effectively reduced oxidative stress, promoted collagen synthesis, and inhibited matrix degradation. Therefore, compound **2** appears to be a promising candidate for preventing skin aging.

The effects of ginsenoside Rf (**2**) on the skin have been relatively less studied. Other ginsenosides, like ginsenoside Re, have been extensively researched for their anti-inflammatory and antioxidant properties that benefit skin health, but few studies have directly focused on ginsenoside Rf. More research is needed to confirm the skin benefits of ginsenoside Rf and to compare it with better-known similar compounds.

### 2.5. Effect of Compound ***2*** on ROS and Pro-Inflammatory Cytokines Secretion in TNF-α-Treated NHDFs

**Figure 6:**
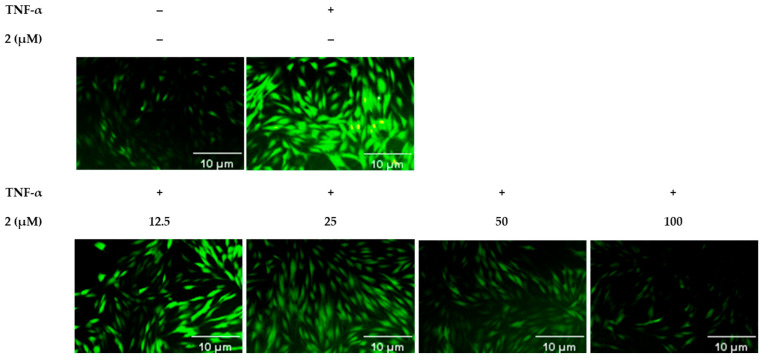
Effects of compound **2** on intracellular ROS accumulation. Cells were stained with dichlorofluorescein diacetate (DCFDA), and fluorescence images were obtained using a fluorescence microscope.

When cells were exposed to TNF-α, a significant increase in fluorescence was observed, indicating elevated levels of reactive oxygen species (ROS). However, upon treatment with compound **2**, a dose-dependent decrease in fluorescence intensity was noted (Figure 6). This reduction suggests that compound **2** effectively counteracts the oxidative stress induced by TNF-α. The ability of compound **2** to reduce ROS production indicates its potential therapeutic role in minimizing skin damage linked to oxidative stress, which is often aggravated by external factors like UV radiation.

**Figure 7:**
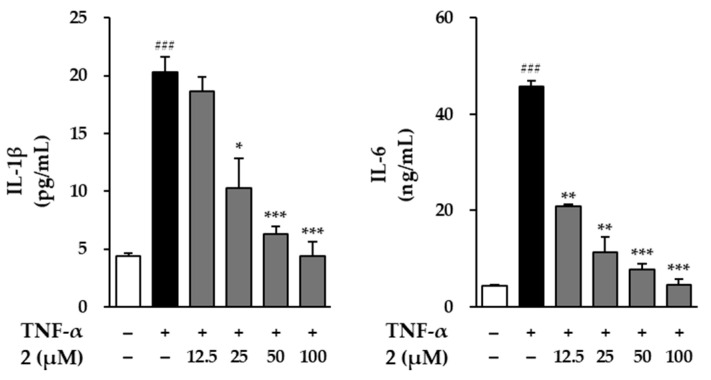
Anti-inflammatory effects of compound **2** on IL-1β, and IL-6 secretion. The levels of IL-1β and IL-6 in the cell supernatants were measured using an ELISA kit. The results are presented as fold increases relative to the control, derived from two independent experiments, and are shown as the mean ± SEM. Statistical significance is indicated as ^###^ *p* < 0.001 vs. vehicle control, and * *p* < 0.05, ** *p* < 0.01, *** *p* < 0.001 vs. TNF-α-stimulated control.

UV radiation stimulates the production and release of inflammatory mediators in various skin cells. This inflammation leads to oxidative damage of cellular components, including proteins, lipids, and carbohydrates, which accumulate within the dermal and epidermal layers, contributing to photoaging [30]. To investigate potential anti-inflammatory effects, we examined the impact of ginsenoside Rf (**2**) on the secretion of interleukin 6 (IL-6) and interleukin 8 (IL-8) in TNF-α-treated natural killer cells (Figure 7). Following TNF-α exposure, IL-6 secretion levels increased significantly to 7.29 ± 0.02 ng/mL. However, treatment with compound **2** at all tested concentrations led to a marked reduction in IL-6 secretion, with the 25 µM concentration showing the most significant effect, reducing IL-6 levels to 5.01 ± 0.01 ng/m. In the case of IL-8, TNF-α treatment increased secretion to 0.85 ± 0.01 ng/mL. Compound **2** treatment at 12.5 and 25 µM successfully reduced IL-8 secretion to 0.70 ± 0.03 and 0.62 ± 0.02 ng/mL, respectively, demonstrating an anti-inflammatory response.

### 2.6. Effect of Compound ***2*** on Inflammatory Response in TNF-α/IFN-γ-Treated NHEKs

**Figure 8:**
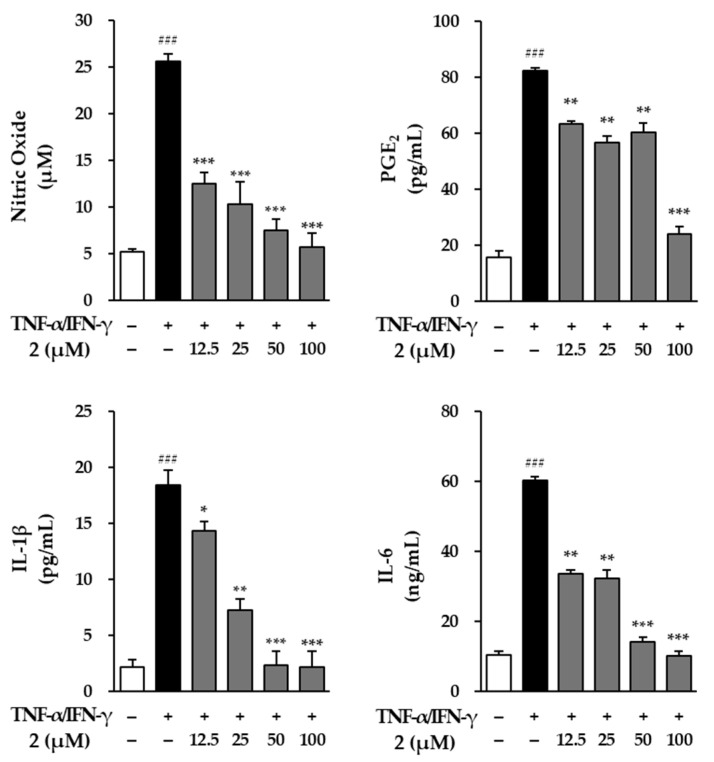
Effects of compound **2** on NO, PGE_2_, IL-1β, and IL-6 secretion in TNF-α/IFN-γ-stimulated normal human epidermal keratinocytes (NHEK). NO was measured using the Griess reaction reagent. The levels of PGE2, IL-1β, and IL-6 in the cell supernatants were measured using an ELISA kit. The results are presented as fold increases relative to the control, derived from two independent experiments, and are shown as the mean ± SEM. Statistical significance is indicated as ^###^ *p* < 0.001 vs. vehicle control, and * *p* < 0.05, ** *p* < 0.01, *** *p* < 0.001 vs. TNF-α/IFN-γ-stimulated control.

The effects of ginsenoside Rf (**2**) on key inflammatory mediators, including NO, PGE2, IL-1β, and IL-6, were evaluated in NHEK cells stimulated with TNF-α and IFN-γ (Figure 8). To accurately measure PGE_2_ levels in the cell culture supernatants, both the Griess reagent method and ELISA kits were used, ensuring precise quantification of this inflammatory marker.

Exposure to TNF-α and IFN-γ led to a significant increase in the production of NO, PGE2, IL-1β, and IL-6, compared to untreated control cells. Specifically, NO levels increased sharply to 25.6 ± 0.83 μM (*p* < 0.001) following stimulation, from a baseline of 5.23 ± 0.23 μM in the controls. Treatment with compound **2** effectively countered this rise, with concentrations of 12.5, 25, 50, and 100 μM reducing NO production to 12.5 ± 1.24 μM (*p* < 0.001), 10.3 ± 2.35 μM (*p* < 0.001), 7.53 ± 1.20 μM (*p* < 0.001), and 5.64 ± 1.52 μM (*p* < 0.001), respectively. These results indicate the anti-inflammatory potential of compound **2**.

Similarly, exposure to TNF-α and IFN-γ led to a marked increase in PGE_2_ production, with levels rising from 15.6 ± 2.34 pg/mL in the control to 82.3 ± 1.24 pg/mL (*p* < 0.001). However, compound **2** treatment at varying concentrations effectively reduced these elevated levels. Specifically, a dose of 12.5 μM lowered PGE_2_ to 63.4 ± 0.94 pg/mL (*p* < 0.01), while higher doses of 25, 50, and 100 μM further decreased it to 56.8 ± 2.36 pg/mL (*p* < 0.01), 60.4 ± 3.46 pg/mL (*p* < 0.01), and 24.2 ± 2.46 pg/mL (*p* < 0.001), respectively, indicating NF’s ability to mitigate PGE_2_-related inflammation.

The inflammatory response was evaluated by measuring IL-1β and IL-6 secretion levels. The stimulation of NHEK cells with TNF-α and IFN-γ led to a significant increase in IL-1β, reaching 18.4 ± 1.36 pg/mL (*p* < 0.001), compared to 2.15 ± 0.64 pg/mL in the control group. Treatment with compound **2** resulted in a marked reduction, with concentrations of 12.5, 25, 50, and 100 μM lowering IL-1β levels to 14.3 ± 0.87 pg/mL (*p* < 0.05), 7.24 ± 0.98 pg/mL (*p* < 0.01), 2.35 ± 1.22 pg/mL (*p* < 0.001), and 2.11 ± 1.50 pg/mL (*p* < 0.001). Similarly, TNF-α/IFN-γ stimulation caused a sharp rise in IL-6 production, reaching 60.3 ± 0.92 ng/mL (*p* < 0.001). Compound **2** effectively reduced IL-6 levels at 12.5, 25, 50, and 100 μM, showing significant decreases of 33.5 ± 1.24 ng/mL (*p* < 0.01), 32.3 ± 2.42 ng/mL (*p* < 0.01), 14.2 ± 1.25 ng/mL (*p* < 0.001), and 10.2 ± 1.25 ng/mL (*p* < 0.001), respectively.

Based on the observed results, it is evident that compound **2** has a strong inhibitory effect on key inflammatory mediators in NHEK cells, effectively reducing the production of NO, PGE_2_, IL-1β, and IL-6 that were significantly elevated due to TNF-α and IFN-γ stimulation. The dose-dependent responses indicate that compound 2 not only mitigates the inflammatory response but also exhibits a consistent pattern of efficacy across different markers.

### 2.7. Effect of Compound ***2*** on Skin Barrier in TNF-α/IFN-γ-Treated NHEKs

**Figure 9:**
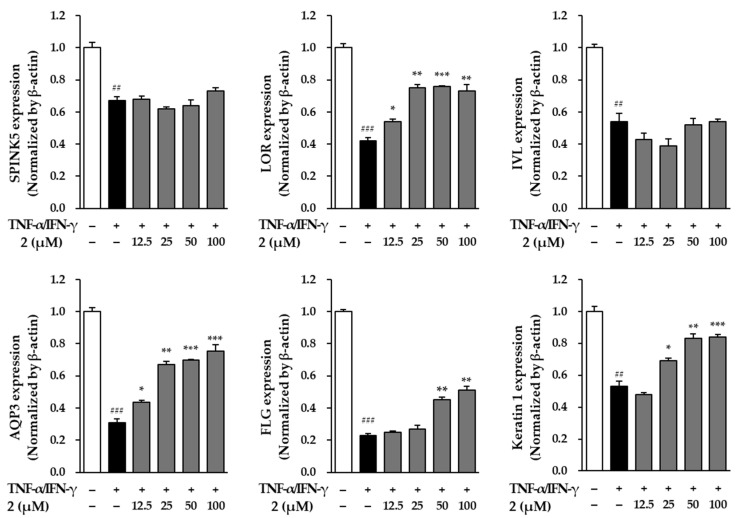
Effects of compound **2** on SPINK5, LOR, IVL, AQP3, FLG, and keratin 1 expression. The expression levels of SPINK5, COLIA1, LOR, IVL, AQP3, FLG, and keratin 1 were measured by qRT-PCR. The results are presented as fold increases relative to the control, derived from three independent experiments, and are shown as the mean ± SEM. Statistical significance is indicated as ^##^ *p* < 0.01 and ^###^ *p* < 0.001 vs. vehicle control, and * *p* < 0.05, ** *p* < 0.01, *** *p* < 0.001 vs. TNF-α/IFN-γ-stimulated control.

The impact of NF on genes linked to skin barrier function was examined in NHEK cells exposed to TNF-α and IFN-γ to replicate inflammatory conditions. The study analyzed key genes involved in maintaining the skin barrier, including serine peptidase inhibitor kazal type 5 (SPINK5), loricrin (LOR), involucrin (IVL), aquaporin-3 (AQP3), filaggrin (FLG), and keratin 1 (KRT1). Gene expression levels were measured using real-time PCR, providing insights into how NF influences skin barrier integrity under stress (Figure 9).

When NHEK cells were exposed to TNF-α/IFN-γ, SPINK5 expression was significantly downregulated, dropping to 0.67 ± 0.03-fold compared to the vehicle group (*p* < 0.001). Treatment with ginsenoside Rf (**2**) did not reverse this effect.

LOR, a key protein for maintaining skin barrier integrity, showed a significant reduction in expression, down to 0.42 ± 0.02-fold (*p* < 0.001), when NHEK cells were exposed to TNF-α/IFN-γ. Notably, treatment with compound **2** at concentrations of 12.5, 25, 50, and 100 μM effectively restored LOR levels, bringing them to 0.54 ± 0.02-fold (*p* < 0.01), 0.75 ± 0.02-fold (*p* < 0.01), 0.76 ± 0.00-fold (*p* < 0.001), and 0.73 ± 0.04-fold (*p* < 0.01), respectively. These findings demonstrate compound **2**’s potential to enhance the expression of essential proteins that support the skin’s physical barrier, even under inflammatory conditions.

Treatment with TNF-α/IFN-γ led to a decrease in IVL expression, lowering it to 0.54 ± 0.05-fold (*p* < 0.01). However, follow-up treatment with NF did not produce significant changes, suggesting that under these conditions, IVL expression may not be highly responsive to the effects of compound **2**.

AQP3, essential for maintaining skin hydration, experienced a significant drop to 0.31 ± 0.02-fold (*p* < 0.001) after exposure to inflammatory cytokines. However, treatment with compound **2** helped recover AQP3 levels, increasing them to 0.43 ± 0.02-fold at 12.5 μM (*p* < 0.01), 0.67 ± 0.02-fold at 25 μM (*p* < 0.05), 0.70 ± 0.00-fold at 50 μM (*p* < 0.05) and 0.75 ± 0.04-fold at 100 μM (*p* < 0.001). These results indicate that compound **2** may help improve skin moisture retention by boosting AQP3 expression even under stress conditions.

Exposure to TNF-α/IFN-γ significantly reduced FLG expression, lowering it to 0.23 ± 0.01-fold (*p* < 0.001). Treatment with compound **2** partially restored FLG levels, increasing to 0.45 ± 0.02-fold at 50 μM (*p* < 0.001) and to 0.51 ± 0.02-fold at 100 μM (*p* < 0.001), indicating a recovery of this protein important for skin hydration and barrier function.

Similarly, keratin 1 expression dropped to 0.53 ± 0.03-fold (*p* < 0.001) under the same conditions, but compound **2** effectively increased keratin 1 to 0.69 ± 0.02-fold at 25 μM (*p* < 0.05), 0.83 ± 0.03-fold at 50 μM (*p* < 0.01) and 0.84 ± 0.2-fold at 100 μM (*p* < 0.001), showing a dose-dependent improvement in keratin maintenance.

The experiments showed that compound **2** treatment effectively restored the expression of key genes related to skin barrier function, such as LOR, AQP3, FLG, and KRT1, in NHEK cells exposed to TNF-α/IFN-γ-induced stress (Figure 9). These findings suggest that NF may help reduce skin barrier disruption and cellular damage caused by inflammation. By enhancing the expression of proteins crucial for skin structure, hydration, and protection, compound **2** emerges as a potential therapeutic option for supporting skin health. Further research could clarify the mechanisms behind its effects and explore applications in skincare and dermatology.

## 3. Discussion

As concerns about skin aging continue to grow in both the health and beauty industries, the use of natural products for anti-aging treatments has garnered significant attention. In the present study, 14 compounds were isolated from the hot water extract of *P. ginseng* roots. The anti-skin aging activity of the extract and the isolated compounds (**1**–**14**) was evaluated in TNF-α induced NHDFs. In addition, the protective effects of compound **2** were further evaluated in TNF-α/IFN-γ-stimulated NHEKs.

In previous studies, ginsenoside Rg2 (**1**) and ginsenoside Rg1 (**4**) have exhibited anti-aging effects in Hs68 and HaCaT cells, respectively. These were related to a reduction in MMP-1 expression and ROS [17,31]. In addition, ginsenoside Re (**5**) and ginsenoside Rb1 (**7**) have been reported to alleviate skin inflammation in animal models [32,33]. For ginsenoside Rf (**2**), anti-melanogenic properties have been shown through the inhibition of the CREB/MITF pathway in Mel-Ab mouse melanocytes and ex vivo human skin, but its protective effects have been less extensively explored [34]. In addition, no skin anti-aging activity of the other compounds has been reported. Moreover, *threo*-guaiacylglycerol-β-ferulic acid ether (**11**), symplocosneolignan A (**12**), 8*E*-decaene-4,6-diyn-1-*O*-β-d-glucopyranosyl-(1″→2′)-β-d-glucopyranoside (**13**), and panaxjapyne D (**14**) were first isolated from *P. ginseng*.

Among the compounds, compounds **2**, **3**, and **7**–**14** showed pronounced antioxidant effects, effectively reducing intracellular ROS levels. This suggests their potential role in alleviating oxidative stress in NHDFs. All the isolates except for compounds **1** and **8** exhibited significant inhibition of MMP-1 production, suggesting their ability to reduce collagen degradation and protect the extracellular matrix from tissue damage. Compounds **2**, **3**, and **12** were particularly effective in enhancing pro-collagen Type I α1 secretion. Their ability to counteract the suppressive effects of TNF-α on collagen synthesis highlights their potential to promote skin health.

Unlike similar compounds like ginsenoside Re (**5**), known for their anti-inflammatory and antioxidant effects, Rf has not been studied as extensively. To address this gap, we investigated the skin health potential of ginsenoside Rf (**2**) more thoroughly. Initially, we screened its ROS-inhibiting effects, which showed promising results. These effects were further confirmed using fluorescence microscopy. Future studies will explore the mechanisms of Rf and its potential applications in skincare.

The findings of this study highlight the potential therapeutic effects of compounds isolated from the hot water extract of *P. ginseng* roots, with a particular focus on compound **2** (ginsenoside Rf). Our research demonstrated that while the extract itself had limited anti-aging activity, several individual compounds effectively mitigated TNF-α/IFN-γ-induced oxidative stress, inflammation, and skin barrier disruption in NHDFs and NHEKs.

Among the tested compounds, ginsenoside Rf (**2**) showed notable multi-targeted effects. It significantly reduced intracellular ROS levels, which is critical in alleviating oxidative stress linked to skin damage and aging. Its efficacy was further confirmed through fluorescence microscopy, highlighting its potential as a strong antioxidant. Additionally, compound **2** was effective in decreasing pro-inflammatory cytokines, such as IL-1β and IL-6, thereby showing promise as an anti-inflammatory agent. This is crucial in conditions where chronic inflammation exacerbates skin damage.

Furthermore, the ability of compound **2** to enhance the expression of essential proteins such as LOR, AQP3, FLG, and KRT1 indicates that it plays a significant role in supporting skin hydration, structural integrity, and overall barrier function. The restoration of these proteins under TNF-α/IFN-γ-induced stress suggests that ginsenoside Rf can help protect the skin from damage caused by external stressors, such as UV radiation.

The spider chart analysis supported these observations by visually comparing the anti-aging efficacy of the compounds. Compound **2** stood out as having robust multi-targeted effects, excelling in reducing oxidative stress, promoting collagen production, and inhibiting matrix degradation. This positions them as promising candidates for future therapeutic applications aimed at combating skin aging and maintaining skin health.

Despite the promising results, this study also emphasizes the need for further research on ginsenoside Rf. While similar ginsenosides, such as Re, are well studied for their anti-inflammatory and antioxidant properties, Rf’s mechanisms and benefits for skin health remain less explored. Future investigations should focus on elucidating the pathways through which ginsenoside Rf exerts its effects, and whether it could be developed into a targeted treatment for various skin conditions.

Overall, this study underscores the therapeutic potential of specific compounds, particularly ginsenoside Rf, in managing skin aging. Their ability to counteract oxidative stress, reduce inflammation, and support skin barrier integrity makes them promising candidates for skincare and dermatological treatments. Further research will be instrumental in harnessing these benefits for broader applications.

## 4. Materials and Methods

### 4.1. General Experimental Procedure

Thin-layer chromatography (TLC) analysis was carried out using silica gel 60 F254 and RP-18 F254S plates (Merck, Kenilworth, MA, USA). To visualize the spotted TLC plate, it was immersed in 20% (*v*/*v*) H_2_SO_4_ (Duksan, Korea) and then heated at 124 °C for 10 min. Column chromatography (CC) was performed to isolate compounds using Diaion HP-20 (Mitsubishi, Tokyo, Japan), Sephadex LH-20 (GE Healthcare, Stockholm, Sweden), silica gel (70–230 and 230–400 mesh, Merck), MCI gel CHP20P (Merck), and RP ODS-A (YMC, Ltd., Kyoto, Japan). Medium-pressure liquid chromatography (MPLC) was implemented with Combi Flash Rf200 (Teledyne Isco., Lincoln, NE, USA) and a pre-packed cartridge, Redi Sep-C18 43 g (Teledyne Isco., Lincoln, NE, USA). Preparative HPLC was operated under the Waters purification system (1525 pump with 996 PDA detector, Waters, Milford, MA, USA) with a Gemini NX-C18 110A column (250 × 21.2 mm i.d., 5 μm, Phenomenex, Torrance, CA, USA), J’sphere ODS-M80 column (250 × 21.2 mm i.d., 5 μm, YMC, Ltd., Kyoto, Japan), and Luna 10 µm C18(2) 100 Å AXIA packed column (250 × 21.2 mm i.d., 10 µm, Phenomenex). The structures of the isolated compounds were elucidated by NMR spectroscopy using JEOL 500 MHz (JEOL, Tokyo, Japan).

### 4.2. Plant Materials

The fresh roots of *P. ginseng* C.A. Meyer (Araliaceae) were obtained from Gyeongdong Market in Dongdaemun-gu, Seoul, South Korea, and were verified by Professor Dae Sik Jang. A voucher specimen (PAGI-2019) of these materials has been stored in the Laboratory of Natural Product Medicine at Kyung Hee University’s College of Pharmacy in Seoul, Republic of Korea.

### 4.3. Extraction and Isolation

Fresh *P. ginseng* roots (40.53 kg) were cut and extracted with 80 L of distilled water at 100 °C in a water bath for 2 h twice. The combined extracts were evaporated at 40 °C to obtain a hot water extract (1.98 kg). The hot water extract was fractionated using Diaion HP-20, resulting in 16 fractions (Fr.1–Fr.16). Fraction F13 was further separated through a series of chromatography methods, including silica gel, Sephadex LH-20, and RP MPLC, leading to multiple subfractions and the isolation of compounds **2**–**14**. Similarly, fractions F14 and others underwent further fractionation and purification steps to isolate compound **1** (Scheme S1).

### 4.4. Cell Viability

NHDFs were seeded into 96-well plates with a clear bottom at a density of 1 × 10^4^ cells per well and allowed to incubate for 24 h to ensure proper cell attachment. Following this initial incubation period, the cells were treated with varying concentrations of the isolated compounds. The treated cells were then incubated for an additional 24 h under standard cell culture conditions in a cell incubator. [35] After the 24 h treatment period, cell viability was assessed using the EZ-Cytox solution, which was added to each well to evaluate the metabolic activity of the cells. The absorbance, indicating cell viability, was measured at 450 nm using a plate reader.

### 4.5. Intracellular Reactive Oxygen Species (ROS)

NHDFs were seeded into black 96-well plates with a flat bottom at a density of 1 × 10^4^ cells per well and incubated for 24 h to allow for cell attachment. To induce starvation conditions, the cells were then maintained in a serum-free medium for another 24 h. Following this period, the cells were treated with various sample compounds for 1 h. After the treatment, they were exposed to 20 ng/mL of TNF-α for 15 min to induce stress response [36]. The cells were then stained with 2′,7′-dichlorodihydrofluorescein diacetate (DCFDA) for 15 min, which allowed for the detection of intracellular reactive oxygen species (ROS).

Subsequently, the supernatant was carefully removed, and the treated NHDFs were washed with PBS to ensure any excess dye was eliminated. Fluorescence was measured using a plate reader, with excitation and emission wavelengths set to 485 nm and 530 nm, respectively, to quantify the levels of ROS present. For more detailed imaging, NHDFs were also plated in 48-well microplates at a density of 2 × 10^4^ cells per well and incubated under standard cell culture conditions for 24 h. Following the same treatment and staining protocol as described, DCFDA-stained cells were immediately observed under a fluorescence microscope, allowing for a visual assessment of ROS levels and distribution within the cells.

### 4.6. Enzyme-Linked Immunosorbent Assay (ELISA)

NHDFs were initially seeded into 48-well plates with a flat bottom at a density of 2 × 10^4^ cells per well and allowed to incubate for 24 h to ensure proper cell attachment and growth. Following this initial incubation period, the culture medium was replaced with a serum-free formulation to induce starvation conditions, mimicking a low-nutrient environment. After maintaining these conditions for 24 h, the cells were treated with various sample compounds for 1 h. Post-treatment, the cells were exposed to 20 ng/mL of TNF-α for an additional 24 h period to stimulate an inflammatory response.

To assess the effects of the treatment, the secretion levels of matrix metalloproteinase-1 (MMP-1) and pro-collagen α1 were measured. This was achieved using enzyme-linked immunosorbent assay (ELISA) kits, which allowed for the specific detection of these proteins in the cell culture supernatants. The optical density, corresponding to the concentration of MMP-1 and pro-collagen α1, was measured using a microplate reader set to an absorbance wavelength of 450 nm.

### 4.7. Spider Chart

Each parameter was divided into four segments based on its respective maximum and minimum values, and each concentration level was assigned a distinct rating. The chart displays the average of these concentrations. The area enclosed by the triangles in the graph represents the relative efficacy.

### 4.8. Statistical Analysis

Data are presented as mean ± standard error of the means (SEMs). Statistical significance was assessed using a one-way analysis of variance (ANOVA) conducted with GraphPad Prism version 5. Tukey’s multiple-comparison test was applied to evaluate differences between groups, with statistical significance set at *p* < 0.05 [37].

## 5. Conclusions

In this work, repeated chromatography was employed to isolate 14 known compounds consisting of seven ginsenosides (**1**–**7**), three phenolics (**8**–**10**), two lignans (**11** and **12**), and two polyacetylene glucosides (**13** and **14**) from the hot water extract of fresh *P. ginseng* roots. *threo*-Guaiacylglycerol-β-ferulic acid ether (**11**), symplocosneolignan A (**12**), 8*E*-decaene-4,6-diyn-1-*O*-β-d-glucopyranosyl-(1″→2′)-β-d-glucopyranoside (**13**), and panaxjapyne D (**14**) were reported for the first time in *P. ginseng*. Ginsenoside Rf (**2**) and other compounds in ginseng root reduced factors associated with skin aging and inflammation, such as ROS and MMP-1. These compounds also show potential as skincare agents that target multiple pathways involved in skin aging and inflammation. Future research should focus on elucidating molecular mechanisms and optimizing formulations for dermatological applications.

## Data Availability

The data of this study are available from the corresponding author upon reasonable request.

## Supplementary Materials

The following supporting information can be downloaded at https://www.mdpi.com/article/10.3390/molecules29225479/s1, Scheme S1. Isolation of compounds **1**–**14** from the hot water extract of fresh *Panax ginseng* roots; Figure S1. Survival of normal human dermal fibroblasts (NHDFs) treated with the hot water extract of fresh *Panax ginseng* roots and compounds **1**–**14**.
