# Supplementary material for: Anti-Aging and Anti-Inflammatory Effects of Compounds from Fresh Panax ginseng Roots: A Study on TNF-α/IFN-γ-Induced Skin Cell Damage
Source: Molecules. 2024 Nov 20;29(22):5479. doi: 10.3390/molecules29225479 (PMC11597146; doi:10.3390/molecules29225479)
Supplement: Supplementary file 1 [file molecules-29-05479-s001.zip › molecules-3303736-supplementary.pdf]

*molecules*

*Supplementary Information*

# Anti-aging and Anti-inflammatory Effects of Compounds from Fresh *Panax ginseng* Roots: A Study on TNF-α/IFN-γ-induced Skin Cell Damage

**Minseo Kang[1,†], Somin Park[2,†], So-Ri Son[2], Yedam Noh[2], Dae Sik Jang[2,*], and Sullim Lee[1,*]**

[1] Department of Life Science, College of Bio-Nano Technology, Gachon University, Seongnam 13120, Republic of Korea
[2] Department of Biomedical and Pharmaceutical Sciences, Graduate School, Kyung Hee University, Seoul, South Korea; allosori@khu.ac.kr (S.-R.S); somin0915@khu.ac.kr (S.M.P)
[†] These authors equally contributed to this work.
[*] Correspondence: dsjang@khu.ac.kr (D.S.J); sullimlee@gachon.ac.kr (S.L.)

*Supplementary S1. Extraction and Isolation*

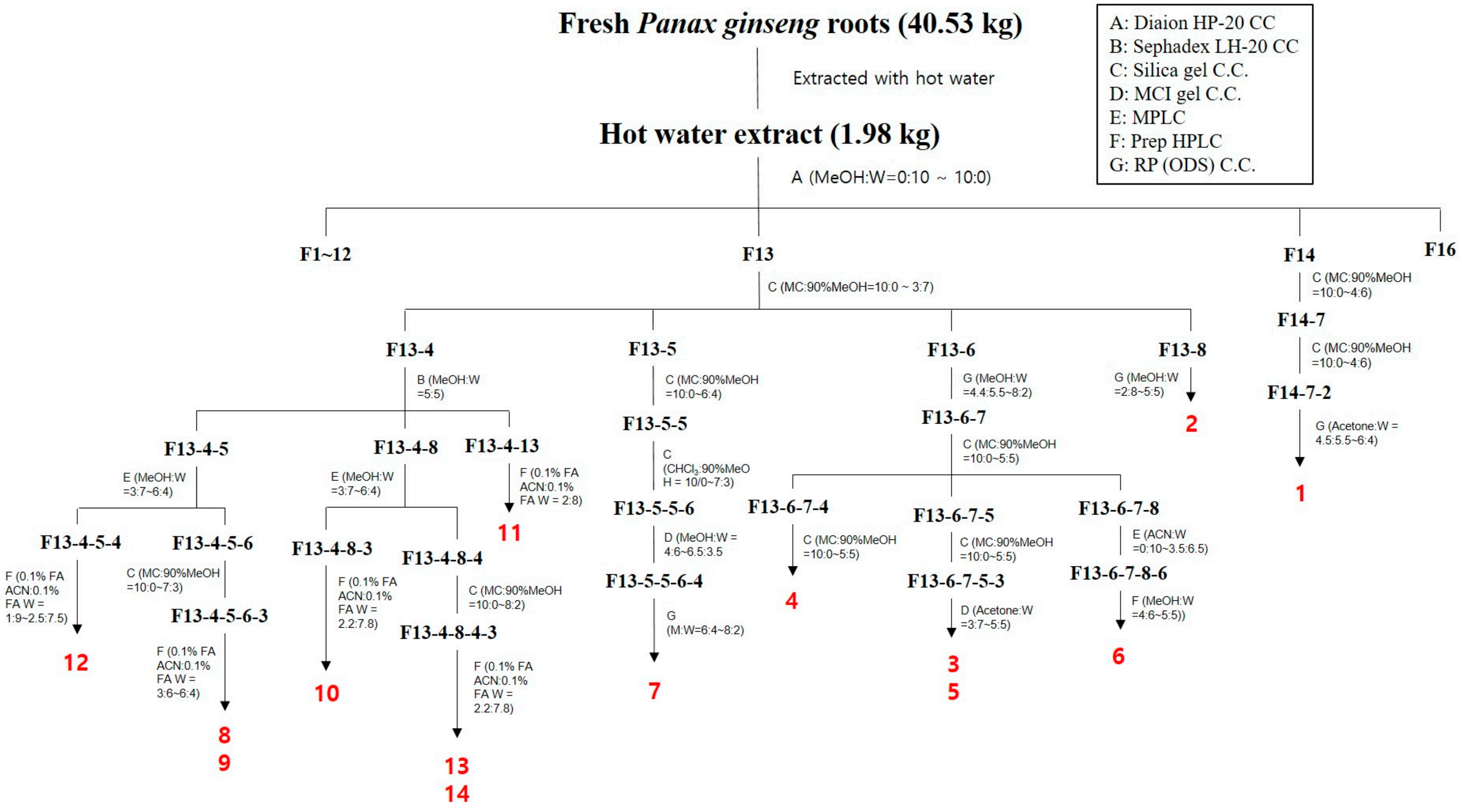


**Scheme S1.** Isolation of compounds **1–14** from the hot water extract of fresh Panax ginseng roots.

Fresh *P. ginseng* roots (40.53 kg) were cut and extracted with 80 L of distilled water at 100 °C in water bath for 2 hours twice. The combined extracts were evaporated at 40°C, to give a hot water extract (1.98 kg). The hot water extract was fractionated by Diaion HP-20 CC (ø 9.8 × 60.0 cm, MeOH/$H_2O$ = 0:10 – 10:0) to yield 16 fractions (Fr1 – Fr16). F-13 was further fractionized by silica gel CC (230–400 mesh, ø 6.5 × 53.0 cm, $CH_2Cl_2$/90% MeOH = 10:0 – 3:7, v/v), resulting in nine subfractions (F13-1 to F13-9). F13-4 was subjected to Sephadex LH-20 CC (ø 1.5 × 60.0 cm, MeOH/$H_2O$ = 5/5, v/v), producing 15 subfractions (F13-4-1 to F13-4-15). F13-4-5 was separated using RP MPLC (43 g, MeOH/$H_2O$ = 3:7 – 6:4, v/v), yielding in 12 subfractions (F13-4-5-1 to F13-4-5-12). For F13-4-5-4, a preparative HPLC with a Gemini column (0.1% formic acid acetonitrile/$H_2O$ = 1:9 – 2.5:7.5, v/v) was employed to isolate compound **12** (1.6 mg). F13-4-5-6 was separated using silica gel CC (230–400 mesh, ø 1.2 × 23.0 cm, $CH_2Cl_2$/90% MeOH = 10:0 – 7:3, v/v), resulting in five subfractions (F13-4-5-6-1 to F13-4-5-6-5). F13-4-5-6-3 was subjected to preparative HPLC with a Gemini column (0.1% formic acid acetonitrile/$H_2O$ = 3.6:6.4, v/v), isolating compounds **8** (2.6 mg) and **9** (1.7 mg). F13-4-8 was separated into eight subfractions (F13-4-8-1 to F13-4-8-8) using RP MPLC (43 g, MeOH/$H_2O$ = 3:7 – 6:4, v/v). F13-4-8-3 (60.0 mg) was subjected to preparative HPLC with a J'sphere column (0.1% formic acid acetonitrile/$H_2O$ = 2.2:7.8), giving compound **10** (1.6 mg). For F13-4-8-4, silica gel CC (230–400 mesh, ø 6.5 × 53.0 cm, $CH_2Cl_2$/90% MeOH = 10:0 – 8:2, v/v) to yield three subfractions (F13-4-8-4-1 to F13-4-8-4-3). F13-4-8-4-3 (20.6 mg) was separated using preparative HPLC with a Gemini column (0.1% formic acid acetonitrile/$H_2O$ = 2.2:7.8, v/v) to afford compounds **13** (3.2 mg) and **14** (4.7 mg). F13-4-13 (29.4 mg) was subjected to preparative HPLC with a J'sphere column (0.1% formic acid acetonitrile/$H_2O$ = 2:8, v/v) to give compound **11** (8.2 mg). F13-5 was fractionated using silica gel CC (230–400 mesh, ø 5.5 × 50.0 cm, $CH_2Cl_2$/90% MeOH) = 10:0 – 6:4, v/v), resulting in seven subfractions (F13-5-1 to F13-5-7). F13-5-5 was further fractionized using silica gel CC (230–400 mesh, ø 5.5 × 50.0 cm, $CHCl_3$/90% MeOH = 10:0 – 7:3, v/v), resulting in eight subfractions (F13-5-5-1 to F13-5-5-8). F13-5-5-6 was separated into six subfractions (F13-5-5-6-1 to F13-5-5-6-6) using MCI gel CC (ø 5.0 × 36.0 cm, MeOH/$H_2O$ = 4:6 – 6.5:3.5, v/v). F13-5-5-6-4 (6.5 g) was subjected to RP ODS-A CC (ø 4.5 × 38.0 cm, MeOH/$H_2O$ = 6:4 – 8:2, v/v) to isolate compound **7** (2.96 g). For F13-6, RP ODS-A CC (ø 4.5 × 38.0 cm, MeOH/$H_2O$ = 4.5:5.5 – 8:2, v/v) was carried out to produce eight subfractions (F13-6-1 to F13-6-8). F13-6-7 was subjected to silica gel CC (230–400 mesh, ø 4.5 × 38.0 cm, $CH_2Cl_2$/90% MeOH = 10:0 – 5:5, v/v), yielding eight subfractions (F13-6-7-1 to F13-6-7-8) and compound **4** (10.64 g). F13-6-7-5 was further fractionized using silica gel CC (ø 3.5 × 40.0 cm, $CH_2Cl_2$/90% MeOH = 10:0 – 5:5, v/v), resulting in three subfractions (F13-6-7-5-1 to F13-6-7-5-3). F13-6-7-5-3 was subjected to MCI gel CC (ø 2.5 × 50.0 cm, acetone/$H_2O$ = 3:7 – 5:5, v/v) to give compounds **3** (26.0 mg) and **5** (6.39 g). F13-6-7-8 was separated using RP MPLC (43 g, acetonitrile/$H_2O$ = 0:10 – 3.5:6.5, v/v), producing 12 subfractions (F13-6-7-8-1 to F13-6-7-8-12). F13-6-7-8-6 was subjected to preparative HPLC with a Luna column (MeOH/$H_2O$ = 4:6 – 5:5, v/v) to yield compound **6** (28.7 mg). For F13-8, RP ODS-A CC (ø 5.0 × 36.0 cm, MeOH/$H_2O$ = 2:8 – 5:5, v/v) was employed to give compound **2** (1.49 g). F14 was fractionized using silica gel CC (70–230 mesh, ø 8.0 × 24.0 cm, $CH_2Cl_2$/90% MeOH = 10:0 – 5:5, v/v) to yield 11 subfractions (F14-1 to F14-11). F14-7 was further fractionized using silica gel CC (230–400 mesh, ø 7.0 × 24.0 cm, $CH_2Cl_2$/90% MeOH = 10:0 – 4:6, v/v), resulting in seven subfractions (F14-7-1 to F14-7-7). F14-7-2 was subjected to RP ODS-A CC (ø 4.5 × 38.0 cm, acetone/$H_2O$ = 4.5:5.5 – 6:4, v/v) to afford compound **1** (601.9 mg).

*Supplementary S2. Effects of the Hot Water Extract and Compounds* ***1-14*** *on Viability of NHDFs*

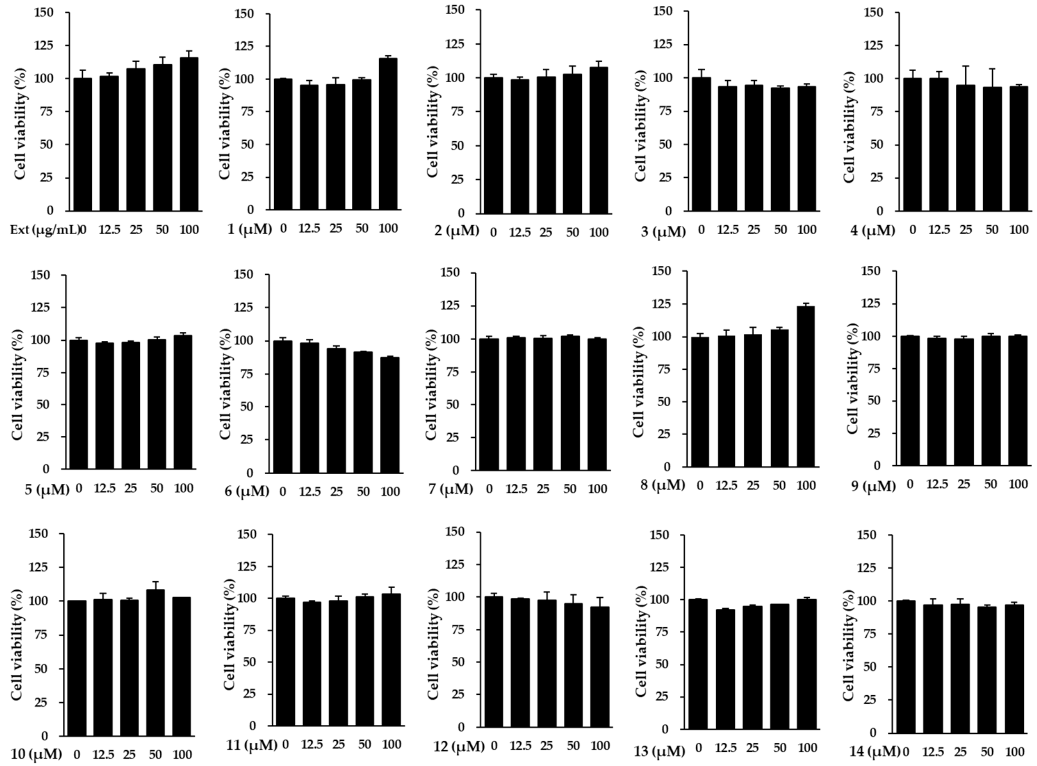

**Figure S1.** Survival of normal human dermal fibroblasts (NHDFs) treated with the hot water extract of fresh *Panax ginseng* roots and compounds **1–14**. NHDFs were seeded at a density of 1 × $10^4$ cells per well in 96-well plates and incubated for 24 hours to allow for cell attachment. After this initial incubation, the cells were treated with various concentrations of each compound for an additional 24 hours. Cell viability was determined using the EZ-Cytox solution, and results are expressed as a percentage relative to vehicle control. Data was collected from three independent experiments, and the results are presented as the mean ± standard error of the mean (SEM).

In this study, we conducted a detailed evaluation of the effects of compounds **1–14** on TNF-$\alpha$-induced damage in Normal Human Dermal Fibroblasts (NHDFs). Prior to assessing their protective effects, we first examined the cell viability of NHDFs treated with each of these compounds to ensure their safety. Each compound was administered to the NHDFs at specific concentrations to determine whether there was any cytotoxic effect (Figure 2). The results indicated that none of the compounds exhibited cytotoxicity, even at the tested concentrations. Based on these findings, we selected a concentration of 100 μM for subsequent experiments to investigate their efficacy against TNF-$\alpha$ induced cellular damage. This approach ensured that the effects observed were not influenced by any potential cytotoxicity of the compounds.
